# Neurological outcome of patients with cryopyrin-associated periodic syndrome (CAPS)

**DOI:** 10.1186/s13023-017-0589-1

**Published:** 2017-02-14

**Authors:** Nafissa Mamoudjy, Hélène Maurey, Isabelle Marie, Isabelle Koné-Paut, Kumaran Deiva

**Affiliations:** 1 Department of Pediatrics, Meaux General Hospital, 77104 Meaux Cedex, France; 2 Assistance Publique-Hôpitaux de Paris, Hôpitaux Universitaires Paris Sud, Department of Pediatrics, Pediatric Rheumatology, National Referral Centre of Auto-inflammatory Diseases, CEREMAI, CHU Bicêtre, University of Paris Sud, Le Kremlin Bicêtre, France; 3 Assistance Publique-Hôpitaux de Paris, Hôpitaux Universitaires Paris Sud, Pediatric Neurology Department and National Referral Center for Neuroinflammatory Diseases in Children and Inserm UMR 1184, Center for immunology of viral infections and autoimmune diseases, CEA, IDMIT, University Paris Sud, 63, rue Gabriel Péri, 94276 Le Kremlin-Bicêtre Cedex, France

**Keywords:** CAPS syndrome, Brain, Children, Outcome, Cognitive

## Abstract

**Background:**

To assess the neurological involvement and outcome, including school and professional performances, of adults and children with cryopyrin-associated periodic syndrome (CAPS).

**Methods:**

In this observational study, patients with genetically proven CAPS and followed in the national referral centre for autoinflammatory diseases at Bicêtre hospital were assessed. Neurological manifestations, CSF data and MRI results at diagnosis and during follow-up were analyzed.

**Results:**

Twenty-four patients (15 adults and 9 children at diagnosis) with CAPS were included. The median age at disease onset was 0 year (birth) [range 0–14], the median age at diagnosis was 20 years [range 0–53] and the mean duration of follow-up was 10.4 ± 2 years. Neurological involvement at diagnosis, mostly headaches and hearing loss, was noted in 17 patients (71%). Two patients of the same family had abnormal brain MRI. *A439V* mutation is frequently associated with a non-neurological phenotype while *R260W* mutation tends to be associated with neurological involvement. Eleven adult patients (61%) and 3 children (50%) underwent school difficulties.

**Conclusion:**

Neurological involvement is frequent in patients with CAPS and the majority of patients presented difficulties in school performances with consequences in the professional outcome during adulthood. Further studies in larger cohorts of children with CAPS focusing in intellectual efficiency and school performances are necessary.

## Background

Cryopyrin-associated periodic syndrome (CAPS) is a rare hereditary periodic fever syndrome with an estimated prevalence in France equal to 1/360 000 [1]. CAPS are caused by dominantly inherited, or de novo, gain of function mutations within the *NLRP3* gene [1, 2]. *NRLP3* encodes cryopyrin, which controls the activation of caspase-1 which in turn catalyses the cleavage of prointerleukin-1β (IL-1β) into the potent proinflammatory cytokine IL-1β [3]. Mutations of *NRLP3* are associated with overactivation of the inflammasome and thus overexpresion of IL-1β. Because of this overproduction of IL-1, a specific treatment of CAPS using anti- IL-1 β monoclonal antibodies such as canakinumab which is a human anti- interleukin-1 β monoclonal antibody are used [4].

The syndrome encompasses a continuum of three diseases, from the mildest familial cold autoinflammatory syndrome (FCAS) to the most severe Neonatal Onset Multisystem Inflammatory Disease (NOMID) also known as Chronic Infantile Neurologic Cutaneous Articular (CINCA) syndrome. The Muckle-Wells syndrome (MWS) has an intermediate phenotype [5, 6]. The clinical manifestations of CAPS such as urticaria-like skin rash, eyes redness, myalgia and sensory deafness, are not specific, if considered separately, and that often leads to a diagnostic delay which compromises the quality of life and exposes the patients to neurosensory complications and renal failure by secondary amyloidosis [5, 7, 8]. Central nervous system manifestations in this syndrome can also occur with various abnormalities on brain MRI [6]. Patients with leptomeningeal or dural enhancement had significantly lower IQ levels than did patients without enhancement. These manifestations are not well known especially by clinicians with a possible underestimation of neurological involvement [9].

The aim of our study is to assess neurological involvement and outcome, including school and professional performances, especially under canakinumab treatment of adults and children with CAPS.

## Methods

### Patients

We analyzed retrospectively data of patients with genetically proven CAPS, followed in the referral center for rare autoinflammatory diseases from January 2002 to January 2015. We identified 24 patients and collected demographic data, age at onset, age at diagnosis and clinical characteristics: fever, skin involvement, musculo-skeletal and ocular manifestations. All patients were followed since their childhood and underwent at least one clinical examination by a neurologist or a pediatric neurologist. Neurological manifestations were defined by any clinical symptoms of neurological involvement: headaches, meningeal syndrome, sensorineural deafness (confirmed by audiograms), optic nerve involvement (based on funduscopic examination), seizures, mental retardation (confirmed by psychometrics tests when available). Headaches were characterized as migraines or not-migraines according to the International Classification of Headache Disorders, 2nd edition. CSF data were collected when available (cells > 5/mm3, proteins >0,4 g/dl, CSF opening pressure > 20 mm H_2_O). Brain MRI (T1, T2, T2 FLAIR, before and after injection of gadolinium, T2* weighted sequences, if available) at diagnostic and during follow-up, were read by two trained pediatric neurologist (NM and KD).

We reported the neurological outcome of these patients based on neurological examination, MRI results and audiometry tests during follow-up. We described their school performances: patients were considered to have learning difficulties if there were academic accommodations, grade repetition, early school leaving, or needing of special education classes. We also described the professional categories of the adult patients with CAPS based on the French National Institute of Statistics (INSEE) classification (Guide des nomenclatures professions et categories socio-professionnelles 2003).

We also analyzed the efficacy of canakinumab on neurological signs. The treatment was considered to be efficient if there was a complete resolution of headaches or if the frequency of these headaches was less than once a month. A partial response was defined if the headaches occured more than once a month. Hearing loss evolution under treatment was evaluated by repeated audiometry tests during follow-up: stable, improvement or worsening.

### Statistical analysis

Statistical analysis was carried out with SPSS version 19.0. Parametric or nonparametric tests (Kruskal-Wallis and Mann–Whitney U tests) were used for continuous measurements as appropriate given normality. Differences were considered significant for *p* values <0.05.

## Results

Twenty-four patients (15 adults and 9 children at diagnosis) with CAPS were analyzed. The median age at disease onset was 0 year (birth) [range 0–14], the median age at diagnosis was 20 years [range 0–53] and the mean duration of follow-up was 10,4 ± 2 years. Two patients had no family history of CAPS.

Skin rash, musculo-skeletal involvement and fever were the most prevalent features as described in Table 1 and neurological involvement at diagnosis was noted in 17 patients (71%). One patient had a mental retardation confirmed by neuropsychological tests (WISC IV), one patient had psychotic disorder. Fourteen (58%) patients had learning difficulties needing academic accommodations (Table 1).

**Table 1:** Characteristics of the patients with CAPS

		*n* = 24	%
Female sex		14	58
Familial history of CAPS		22	92
Genotype	*R260W*	12	50
	*A439V*	4	17
	*T348M*	4	17
	Other mutations	4	17
Age at onset (years)		0 [0–14]	
Age at diagnosis (years)		20 [0–53]	
Mean follow-up duration (years)		10,4 ± 1,94	
Fever		22	92
Skin involvement		24	100
	Urticarial rash	23	96
	Maculopapular rash	17	71
Myalgia		15	62
Arthralgia		21	87
Conjunctivitis		20	83
Neurological signs		17	71
	Headaches	15	62
	Migrainous headaches	4	27
	Chronic daily headaches	8	54
	Hearing loss	10	59
	Optic nerve atrophy	2	8
	Mental retardation	1	0,04
Learning difficulties		14	58
Abnormal brain MRI (*n* = 23)		2	0,09
CSF assays (*n* = 5)	Aseptic meningitis	2	40
	Elevated opening pressure	1	20

The *A439V* mutation was frequently associated with no neurological signs and *R260W* tended to be associated with neurological symptoms (Table 2).

**Table 2:** Genotype correlation with neurological involvement

Genotype	Neurological signs *n* = 20	No neurological signs *n* = 4
*A439V* (%)	1 (25)	3 (75)
*R260W* (%)	12 (60)	0 (0)
*T348M* (%)	3 (15)	1 (25)
Other mutations (%)	4 (20)	0 (0)

**Fig. 1:**
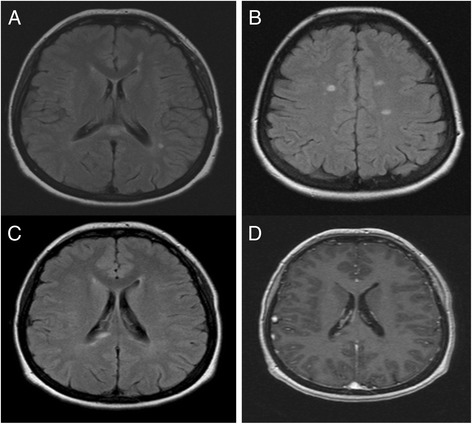
Brain MRI lesions of 52 year-old patient with CAPS before and after treatment. Axial FLAIR brain MRI images showing well limited periventricular (**a**), corpus callosum (**a**) and juxtacortical lesions (**b**). Brain MRI images with Axial FLAIR sequences (**c**) and T1 after gadolinium injection sequences (**d**), after one year of canakinumab treatment, of the same patient showing new FLAIR right paraventricular abnormalities with Gadolinium enhancement

**Fig. 2:**
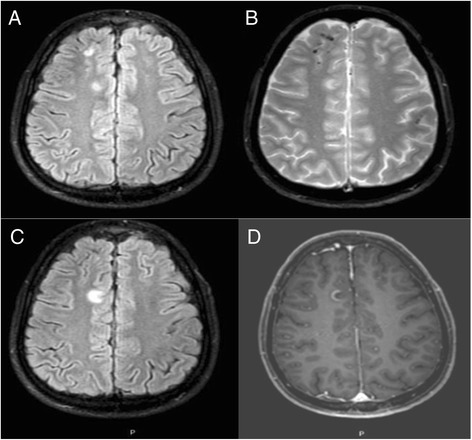
MRI images of 26 year-old patient with CAPS during a relapse with severe headaches. (**a**): Axial Flair images showing T2 FLAIR hypersignal right frontal juxta-cortical lesions with (**b**) hyposignal lesions on T2* weighted sequences suggesting hemosiderin deposits and (**c**) right anterior cingular gyrus T2 FLAIR hypersignal with (**d**) gadolinium enhancement

All but one patient underwent at least one brain MRI before treatment. One brain MRI among 23 was abnormal before IL-1β treatment showing white matter lesions (Fig. 1a, b). The clinical examination of this 45-year-old female who had a heterozygous *R260W* mutation revealed no clinical neurological abnormalities. These MRI lesions were characterized as multiple sclerosis-like lesions. Two lesions appeared on brain MRI after one year of treatment (Fig. 1c, d). After that, MRI remained stable as well as her clinical neurological examination and the patient was already reported in the literature by our group [10]. The MRI of one 25 year-old patient showed T2 lesions after seven years of canakinumab treatment and with no clinical consequences (Fig. 2). Lesions were compatible with a vasculitis. These 2 patients are from the same family and carry the same mutation *R260W*.

Five patients (21%) underwent a CSF analysis; the opening pressure of the CSF was measured for 3 of them: 2 patients had an aseptic meningitis and the CSF opening pressure of one patient was elevated at 36 mm H_2_O. These two patients had not migraine-headaches.

The patients who had multiple sclerosis-like MRI lesions underwent after 8 months of imaging, a CSF analysis, which was normal.

Twenty-one patients (87%) are treated with canakinumab (starting at a dose of 150 mg every 8 weeks for adults and 2 mg/kg for children administrated as a subcutaneous injection). Treatment was not started in two children (9 and 4 years old) as their symptoms had very low impact in their everyday life. One 42 year-old female patient still refuses the treatment.

Fourteen (82%) of the treated patient described improvement of their headaches: a complete resolution of headaches in 12 patients (80%) and partial resolution in 2 (13%) was observed. One patient had no improvement of his migrainous headaches under treatment. The elevated opening CSF pressure of one patient, normalized two years after treatment onset.

Eight (80%) patients reported improvement (*n* = 3) or stabilization (*n* = 5) of their hearing loss proven by audiometry during follow up.

**Fig. 3:**
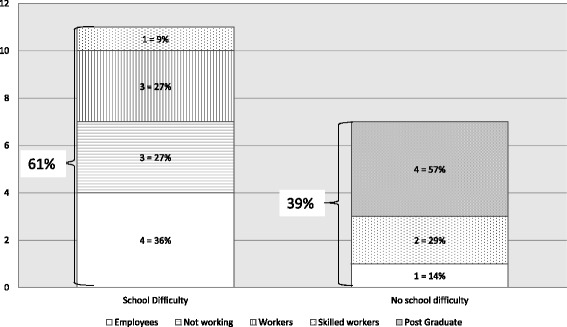
Professional categories of adults with CAPS (*n* = 18)

At follow-up, 6 patients were less than 18 years old, among them 3 (50%) have learning difficulties needing school accommodations, and 3 (50%) have no school difficulties. The professional categories of adult patients (n = 18) are described in (Fig. 3). Eleven adult patients (61%) underwent school difficulties, at follow-up 3 have no jobs, 3 are workers, 1 is a skilled worker and 4 are employees. Among adults who had no school difficulties, 4 are post-graduate students, 1 is an employee and 2 are skilled workers (Fig. 3).

## Discussion

In this retrospective observational study, neurological involvement concerns 71% of patients with CAPS, mostly with headaches and neurosensorial hearing loss. Canakinumab treatment confirms its efficacy in neurological symptoms in 76% of treated patients [4]. The most important findings in our study is the impact of CAPS on school performances: 50% of patients under 18 years old with CAPS have learning difficulties needing school accommodations, and 61% of adult patients had school difficulties with probable consequences in their professional careers.

In the largest CAPS study based on Eurofever registry (*n* = 136), neurological manifestations were observed in 40% and is mostly represented by headaches (70%), papilledema (52%), meningitis (26%), hydrocephalus (18%), mental retardation (16%), seizures (4%) [9]. Hearing loss which is secondary to a cochlear inflammation represented 42% and was not included in the neurological symptoms, which can explain the underestimation of neurological involvement [9]. In our study, we confirmed that headaches and hearing loss were the most prominent neurological symptoms,

The mechanism of headaches in patients with CAPS is not well known. In our study, 4 patients (27%) had migrainous headaches and 2 patients with non-migrainous headaches had an aseptic meningitis and one of them had a high opening CSF pressure showing an intracranial hypertension which can explain the headaches [11]. Only 21% of our patients underwent CSF analysis and CSF opening pressure was measured only for 2 patients. Unfortunately, CSF assays was not exhaustive and intracranial hypertension and or chronic meningitis might be under-estimated. A high level of pro-inflammatory cytokines including IL-1β in patients with migraines had been described [12]. This may be relevant to understand the physiopathological mechanism of headaches in CAPS and can explain canakinumab efficacy which was complete for 2 of 4 patients with migrainous headaches.

Fifty nine percent of the patients with CAPS have a hearing loss. The physiopathology is not well known, it has been observed that some children with CINCA had a cochlear inflammation demonstrated on MRI, after IL-1 receptor antagonist treatment there was an improvement of cochlear enhancement [6]. In our study, no cochlear inflammation has been described on MRI but this imaging was not specifically performed for inner ear disease, and cochlear abnormality on MRI may have not been seen.

Before any anti- IL-1β treatment, one patient had white matter lesions with demyelination as it may be seen in multiple sclerosis without any neurological sign (except for chronic headaches) unlike the only other CAPS patient described in literature with demyelinating disease who had a hemiparesia [13]. In MS, it has been suggested that brain lesions may also be induced following inflammatory synaptopathy and neurodegeneration caused by IL-1β [14] and a similar mechanism may explain brain lesions in patients with CAPS syndrome. Recent studies suggested that IL-1 receptor antagonist (IL-1Ra) could cross the blood–brain barrier and have a neuroprotective effects in rodent model as well as in patients with ischemic stroke or subarachnoid hemorrhage brain hemorrhage [15]. Under anti-IL-1β, in one patient with white matter lesions before the start of treatment, 2 new lesions appeared after one year of treatment. Thereafter, all brain MRI of this patient remained stable suggesting that the lesion may eventually be associated with the disease and treatment may have an effect on long term.

Although we could not perform statistical test du to small numbers of included patients, we have observed that *A439V* mutation is frequently associated with a non-neurological phenotype as previously published [9]. Interestingly, we also noticed that *R260W* mutation, tend to be associated with neurological involvement. Moreover 2 members of the same family with this mutation had brain MRI lesions suggesting that brain lesions in these patients may be linked to the disease. Larger studies are needed to detect any specific pattern of these lesions.

Our study described the neurological outcome under anti- IL-1β treatment with a mean duration of follow-up of 10,4 years. Canakinumab had shown its sustained efficacy on neurological symptoms: 82% improved their headaches under treatment, and 80% improved or stabilized their hearing loss. In our study the median age at onset is birth and the median age at diagnosis is 20 years. There is an important delay of diagnosis and therefore a delay for treatment, already noted in previous studies [9]. This highlights the importance of recognizing the clinical features of this rare syndrome for neonatologists, pediatrician and pediatric neurologist in order to initiate the specific treatment and reverse systemic symptoms as well as neurological symptoms and prevent progression of them, especially headaches and hearing loss.

Our study focused on school performances and professional career during follow-up. Although there is only one objective mental retardation, school difficulties are frequent in children with CAPS. The cognitive difficulties may be under-estimated: only 4 patients underwent psychometric tests. The impact of CAPS in school and professional performances is multifactorial: chronic pains, school absences, sick leaves are also implicated. A recent study showed on 7 patients in a prospective monocentric study that canakinumab had not only a sustained effect on quality of life, but also allowed dramatic changes in social and work lives [16]. The benefit of canakinumab treatment in allowing a professional life was one of their most important observations; no patients were out of work during the follow-up (4.8 ± 0.8 years). The gain in vitality scores permitted them to enjoy a social life after work and physical activity, which were impossible without treatment. Psychometric tests, neuropsychological evaluation, neuropediatric follow-up is not systematic for children with CAPS, although the learning difficulties are frequent in these patients as shown in our study.

## Conclusion

We provided a detailed description of the neurological involvement, which is frequent in adults and children with CAPS. Canakinumab seems to have changed the general outcome of these patients as well as the neurological outcome. Further studies in larger cohorts of children with CAPS focusing in intellectual efficiency and school performances are necessary. Because of the frequency of school difficulties shown in our study in children with CAPS, the role of the pediatric neurologist in the muldisciplinary follow-up of these patients is very important and regular neuropsychological evaluations and neurological following are advisable for children with CAPS.

## Abbreviations

CAPS: Cryopyrin-associated periodic syndrome
CINCA: Chronic Infantile Neurologic Cutaneous Articular syndrome
CSF: Cerebro-spinal fluid
MRI: Magnetic resonance imaging
MWS: Muckle-Wells syndrome
NOMID: Neonatal Onset Multisystem Inflammatory Disease
